# Levels of the Thiocyanate in the Saliva of Tobacco Smokers in Comparison to e-Cigarette Smokers and Nonsmokers Measured by HPLC on a Phosphatidylcholine Column

**DOI:** 10.3390/molecules24203790

**Published:** 2019-10-21

**Authors:** Jolanta Flieger, Justyna Kawka, Małgorzata Tatarczak-Michalewska

**Affiliations:** Department of Analytical Chemistry, Medical University of Lublin, Chodźki 4A, 20−093 Lublin, Poland; justynakawka@umlub.pl (J.K.); malgorzatatatarczakmichalewska@umlub.pl (M.T.-M.)

**Keywords:** phosphatidylcholine column, thiocyanate, human saliva, tobacco smokers, e-cigarette smokers, sustainable chemistry

## Abstract

The aim of the study was to estimate the thiocyanate levels in saliva of cigarette smokers in comparison to e-cigarette smokers and nonsmokers. To improve our understanding of the influence of smoking on the oral level of thiocyanate, we conducted an assessment of human saliva, in 24 individuals (eight tobacco smokers, eight e-cigarette smokers, and eight nonsmokers). High-Performance Liquid Chromatography with ultraviolet detection (HPLC-UV) using a unique phosphatidylcholine column was applied in this assay. Thiocyanate ion was detected directly by its absorbance at 210 nm. The method presents a new application of the IAM (Immobilized Artificial Membrane) column for quantification of inorganic anions. The whole process meets the criteria of green chemistry because it was carried out without the use of organic solvents. For compensating matrix effects, an eight-point standard addition protocol was used to quantify the thiocyanate level in saliva samples. The calibration graphs were linear in the range of 5–100 mg L^−1^ with a correlation coefficient higher than 0.99. The thiocyanate concentrations in the saliva of tobacco smokers, e-cigarette smokers, and nonsmokers were found in the range of 121.25–187.54 mg L^−1^, 121.24–244.11 mg L^−1^, 33.03–79.49 mg L^−1^, respectively. The present study indicates an obvious statistically significant elevation in salivary thiocyanate level in tobacco smokers in comparison to nonsmokers. The phosphatidylcholine-based stationary phase proved to be suitable for the detection and quantification of the thiocyanate ion. The salivary thiocyanate levels in e-cigarette smokers were not significantly different in comparison to tobacco smokers but higher if compared to nonsmokers. The criterion for statistical significance was *p* < 0.05.

## 1. Introduction

Composition of tobacco smoke is a mixture of gases such as carbon monoxide and hydrogen cyanide. A main metabolic product of cyanide is thiocyanate (SCN^−^) belonging to essential markers of smoke inhalation besides carbon monoxide, nicotine, and cotinine. SCN^−^ is secreted in saliva and has rather a long half-life of 10−14 days [1,2]. Salivary thiocyanate concentration in nonsmokers ranges from 0.5 to 2 mM with an average of 1 mM whereas smokers may have salivary concentration range as high as 6 mM [3,4,5]. It has been already proven that the elevated level of SCN^−^ in the saliva of smokers has a property to induce cancerous changes directly in the epithelium. An excessive cancer risk occurs through the nitrosation process [6,7].

Currently, the e-cigarette has appeared to be a healthier smoking alternative. E-cigarettes have attracted millions of users around the world since they first appeared on the Chinese market in 2004.

In 2018, results were published indicating that nitrosamines, present in e-cigarettes, can damage DNA, and there is a possibility that e-cigarette smoke may contribute to lung and bladder cancer and heart disease [8]. Taking into consideration the adverse effects of tobacco consumption, the present study was conducted aiming to investigate the salivary SCN^−^ levels in smokers and healthy volunteers to compare and correlate these levels with e-cigarettes users. Salivary thiocyanate is responsible for various neurological disorders (amblyopia, infant squint in children of smoking mothers) and endocrine diseases (an increase in the frequency of nodular goiter) [9,10,11]. Increased salivary SCN^−^ levels can lead to delayed wound healing [12,13]. Determination of SCN^−^ levels in saliva is the most commonly used biochemical test to determine the prevalence of tobacco consumption among adolescents [14].

Saliva as a biological material can be used as a non-invasive, uncomplicated diagnostic tool, useful for disease screening. Obtaining a saliva sample is a rapid, simple, and most importantly painless process. Thus far, saliva has been used to diagnose different autoimmune diseases such as diabetes, cardiovascular diseases, dental caries, and other oral diseases [15,16,17].

The most commonly applied procedure of the thiocyanate quantification is a photometric method published by Degiampietro et al. [18]. This protocol is based on the FeSCN^2+^ complex formation, characterized a deep red color, which is measured close to its absorption maximum at 492 nm or at 447 nm wavelengths. Then SCN^−^ concentration was further calculated using the Lambert-Beer relationship [19,20]. Many methods are routinely dedicated for the determination of the anionic species in saliva, such as ultraviolet–visible (UV–Vis) spectrophotometry [21,22,23,24], flow injection analysis [25], ion chromatography [26,27], high performance liquid chromatography [28,29], capillary electrophoresis (CE), including: micellar electrokinetic chromatography using zwitterionic micelles [30], capillary isotachophoresis [31], and capillary zone electrophoresis (CZE) [32,33]. Among them, there are only a few describing determination of SCN^−^ levels in human saliva [30,31,33].

The purpose of this study was to evaluate the validity of salivary thiocyanate levels in samples of tobacco smokers, e-cigarette smokers, and nonsmokers. An accurate, fast, and simple high-performance liquid chromatography (HPLC) with a diode-array detector (DAD) method was developed. Measurements have been performed on phosphatidylcholine immobilized artificial membrane (IAM) column. This HPLC column has been designed for the prediction of drug-membrane interactions. There is evidence that the retention measured on this column correlates to traditional in-vitro methods such as Caco-2 cell line cultures, intestinal tissue, or liposome assays [34,35,36]. However, it has been previously discovered that inorganic anions are also able to retain on a phospholipid monolayer by Hu et al. [37]. They claimed that owing to the presence of the quaternary ammonium groups on the surface, an anion-exchange mechanism governs the retention process [37]. Recently, this column has been applied to determine levels of iodide in mineral water samples as well as for determination of nitrite and nitrate in human saliva [38,39]. 

Here, we developed a new high-performance liquid chromatography method to quantify the thiocyanate, in human saliva on phosphatidylcholine column for the first time. The method covered a convenient and environmental safe and simple sample preparation approach without derivatization step (samples were processed by deproteinization by boiling combined with centrifugation). The standard addition method was used for quantification. The total HPLC analysis time was short (only 5 minutes), the mobile phase did not contain an organic solvent, considering the fact that HPLC-DAD instrumentation is the most readily available and widespread instrumentation available in most laboratories, and the applied conditions are suitable for enabling analysis on large scale. The advantages of the method developed compared with the methods designed previously for quantification of thiocyanate cover the following: (i) using universal HPLC-DAD instrumentation, (ii) the new application for phosphatidylcholine column used mainly for the chromatographic estimation of the membrane permeability of small molecule drugs and to mimic the interaction of analytes with biological membranes thus far, (iii) simple, green sample preparation method without organic solvents, and without the need for derivatization steps, (iv). short analysis time (shorter than 5 min) achieved by the use of the mobile phase containing NaCl in water, and (v) the beneficial LOD value 15.97 µg L^−1^ which is much lower in comparison to traditional colorimetric analyses based on König synthesis of pyridine-derived dyes (LOD 2 µmol L^−1^ = 116 µg L^−1^) [40].

Furthermore, overload conditions which are very critical for the separation on phosphatidylcholine column have been established in relation to thiocyanate ions for the first time.

## 2. Results and Discussion

### 2.1. HPLC Conditions

IAM.PC.DD2 stationary phases were prepared by immobilization of phosphatidylcholine (PC) on propylamino-silica skeleton where the end-capping procedure of the free propylamino residues has been performed by methylglycolate. An immobilized phosphatidylcholine molecule contains groups that may be positively and negatively charged, whereby the surface charge (or zeta potential) is influenced by certain circumstances [41].

A phospholipid column has a significant impact on the retention as a result of the mass transfer and interaction of charged analytes. The phosphate group linked to the glycerol backbone of PC is negatively charged, whereas the positively charged group belongs to the choline moiety. More precisely, the positive charge of quaternary ammonium group is concentrated on a single nitrogen atom, which is favorable for electrostatic interaction with anions above the isoelectric point of PC (pI = 4.13) [41]. Thus, PC is positively ionized within a narrow pH range. That is why it can be categorized as a weak ion exchanger possessing the ability to act as an ion exchange matrix. It has already been proven that anions are retained on a phospholipid monolayer on the basis of an anion-exchange process, owing to net surface charge [37]. Therefore, the retention and elution of ions can be routinely controlled by adjusting the mobile phase salt concentration. When the ionic strength of the mobile phase increases, salt ions compete for binding to the matrix, displacing the target analyte.

**Figure 1:**
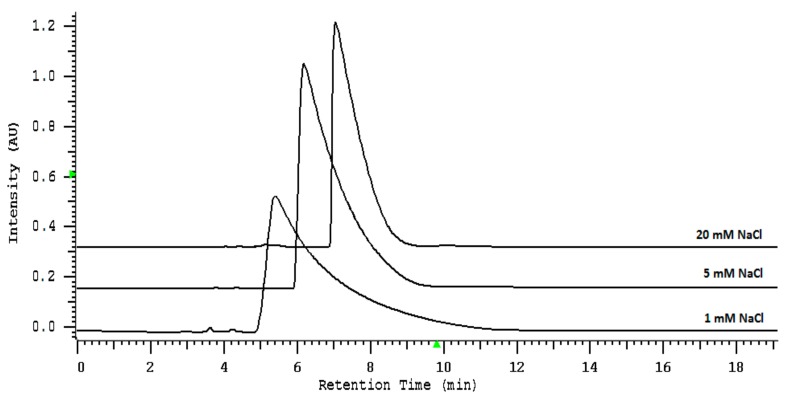
The influence of NaCl concentration in the mobile phase in the range of 1 to 20 mM for the thiocyanate retention, the peak symmetry, and the efficiency. The column: IAM.PC.DD2 Regis HPLC Agilent Technologies, flow rate of the mobile phase: 0.5 mL/min, injected volume: 10 μL, and analytical wavelength: 210 nm.

To establish the optimal salt concentration in water as the mobile phase, thiocyanate was eluted by the mobile phase containing increasing amounts of sodium chloride (NaCl). The effect of NaCl concentration on the peak shape and retention behavior is illustrated on the graph on Figure 1.

The obtained results show that the retention time increases up to 8 min and then remain constant with increasing concentration of NaCl in the mobile phase. A concentration of 20 mM was chosen in subsequent experiments since it provided good retention, symmetrical peak shape, and sufficient separation from matrix constituents.

### 2.2. Loadability Studies

**Figure 2:**
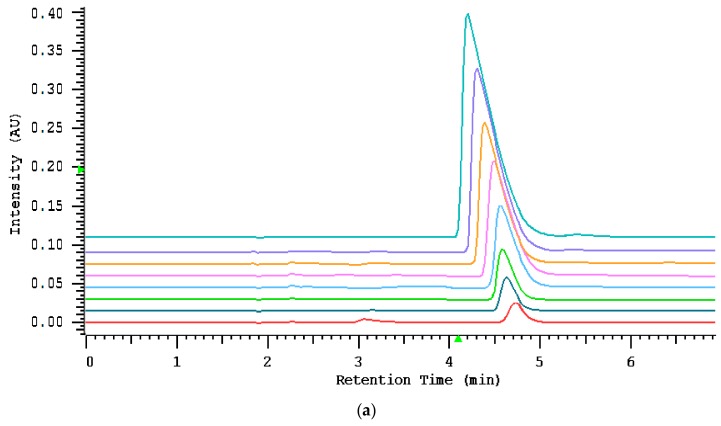

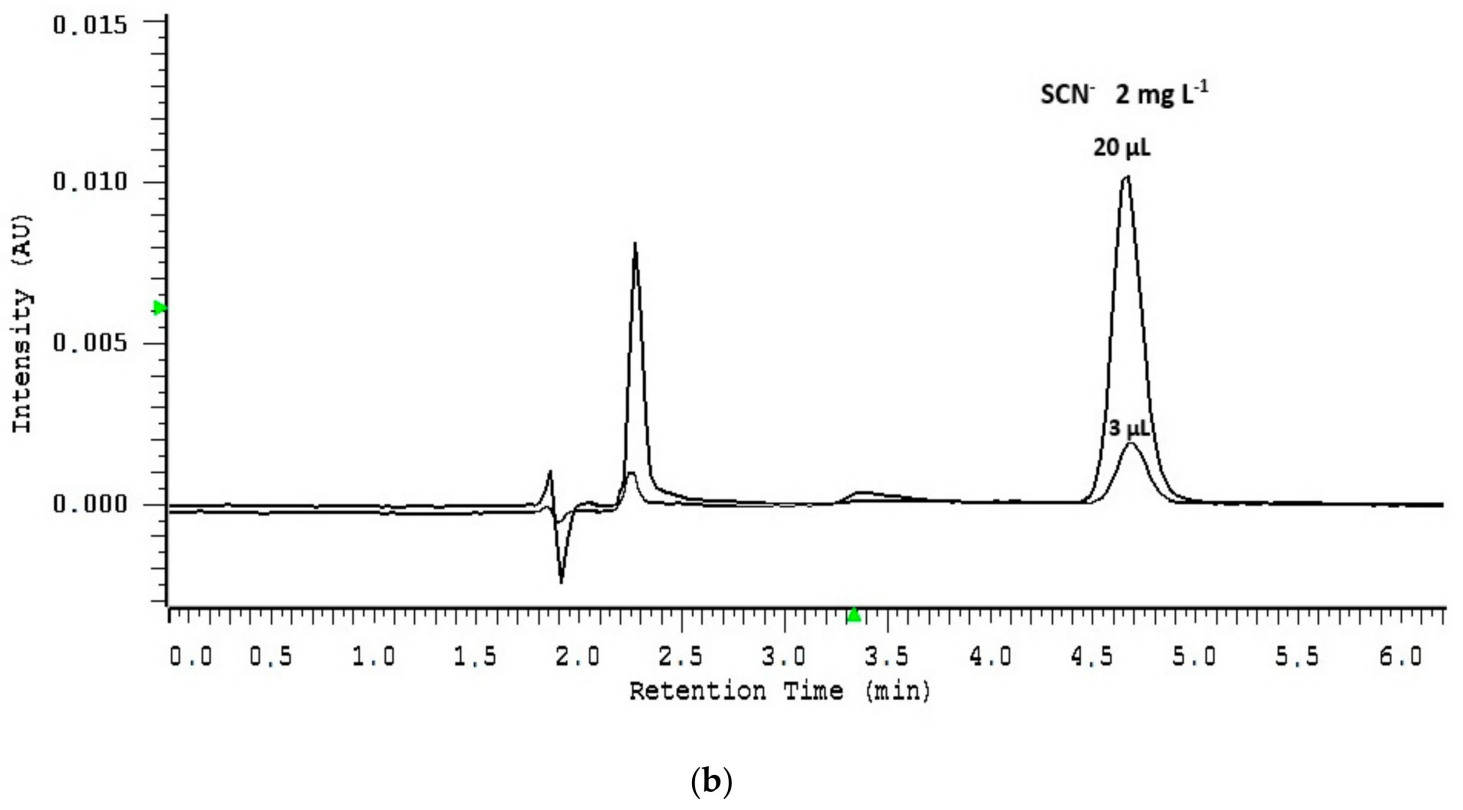
(**a**) Loadability study using a phosphatidylcholine column (IAM.PC.DD2 Regis HPLC) with total sample loads of 10; 15; 25; 50; 80; 100; 150; 200 mg L^−1^ subsequently from the bottom to the top. Flow rate: 1 mL/min; detection: UV absorbance at 210 nm; injection volume: 10 µL. (**b**) The column volume loadability. The first line represents an injection volume of 3 µL, the second line refers to the volume of 20 µL. The sample concentration is 2 mg L^−1^.

A column can be overloaded by increasing the sample concentration as well as the injected volume. The test to confirm column overload was performed by increasing the amount of sample while maintaining a constant injection volume. As can be seen on Figure 2a, the classic symptoms of concentration overload were observed such as the retention time reducing with an increasingly steep front edge of the peak. Under volume overload conditions, concentration remained constant, but the injection volume was increasing constantly. Increasing sample volume resulted in only peak broadening, whereas retention times and peaks symmetry remained at an almost constant level. The peak shapes obtained by volume overload are shown in Figure 2b.

Using the above experimental data, the loadability of the column representing the maximum sample size (mass and volume) can be approximately estimated. The parameters influencing the mass loadability of a column are the following: particle diameter (dp), adsorbent surface (As), packing density (d), partition coefficient (K), column length (l), column radius (r), and appropriate constants (C1, C2). In turn, the volume loadability linearly mainly depends on the dead volume (V_0_) of the column used, on the capacity factor values (*k*), and on the separation efficiency of the column (*n*). Moreover, the column loadability could be controlled by the flow rate. There are at least several definitions of the loadability [42,43,44,45]; however, an empirical determination is more likely to be used in practice. In particular, when the concentration increased from 10 ppm to 200 ppm with the total mass load ranging from 0.1 µg to 2 µg, a loss of peak symmetry occurred between the 0.15- and 0.25-µg load. In turn, the column volume loadability was improved when we performed the experiments loading smaller than 10 µL sample. In fact, we were able to load 0.225 µg of thiocyanate into a column at a volume of 10 µL and still obtain a symmetrical peak with the appropriate plate number.

### 2.3. Validation Studies

To prove that the proposed analytical method ensures for required criteria for various applications, validation of the method was performed according to the United States Food and Drug Administration (FDA) issued guidelines on analytical method validation [46]. However, these guidelines are designed mainly for the validation of exogenous compounds. The quantification of endogenous compounds is still a challenge, because of the lack of blank matrix. More recently, the FDA issued a section for the quantification of endogenous compounds [47]. The FDA guidelines recommended preparing calibration standards in the same biological matrix as studied samples. Various approaches could be used to eliminate provide this condition as background subtraction, the standard addition method, neat solutions, artificial matrices of biological fluids, stripped matrices, or surrogate analytes.

The thiocyanate in human saliva was quantified using the method of standard addition, because blank matrices are not available. In this method, every sample was divided into aliquots of equal volumes. All aliquots were separately spiked with varying amounts of the analyte standards to construct a calibration curve. The sample concentration was determined as the negative x-intercept of the standard calibration curve. The standard addition method ensured direct quantitation of endogenous analytes without subtraction of background peak areas as well as the use of the exact same matrix of every examined sample.

#### 2.3.1. Assay Characteristics

Assay accuracy was calculated using three spiked saliva samples in different concentration levels (5, 50, 100 mg L^−1^), covering the entire range of salivary thiocyanate levels in diluted samples. We found mean accuracies (± S.D.) between 90.04 and 97.80%. The recovery of exogenous thiocyanate from saliva was calculated by comparing the peak areas of spiked saliva with those of directly injected saliva solution. Recoveries and their coefficients of variation are presented in Table 1. The mean recovery was > 90% and a small relation to the spiked amount was observed.

Coefficients of variation (CVs) were < 5% for intraday precision and < 9% for interday precision. The limit of detection (LOD) was determined as a signal-to-noise ratio of three. LOD was 15.97 µg L^−1^ for thiocyanate. The lower limit of quantification (LOQ) for exogenous thiocyanate was determined to be 53.24 µg L^−1^ with a coefficient of variation (CV%) < 5% both for within-day and between day measurements. 

#### 2.3.2. Stability of Salivary Thiocyanate

Stability of salivary thiocyanate was assessed immediately and after storage for 6, 24, and 48 h at room temperature (20 °C) and in the refrigerator (−18 °C). Immediately frozen samples did not differ significantly in thiocyanate levels compared with samples stored at room temperature (Table 2). As can be seen, regardless of storage conditions, the level of thiocyanates decreased to more than 10% within 48 hours. Therefore, analysis of thiocyanates in the saliva is most reliable if the test is performed immediately after collection.

The effects of sample preparation conditions on the peak area were also tested. Standard stability was measured under various conditions relevant to the developed sample preparation procedure (the mixtures were heated at 100 °C for 15 min, and then centrifuged at 4000 × *g* for 60 min). It was observed that the deviation in the peak surface of the standard is within ±1.4% of the respective nominal value; with CV < 1.2%. The results obtained show that the sample preparation procedure used offers acceptable stability.

### 2.4. Quantitation of Salivary Thiocyanate 

**Figure 3:**
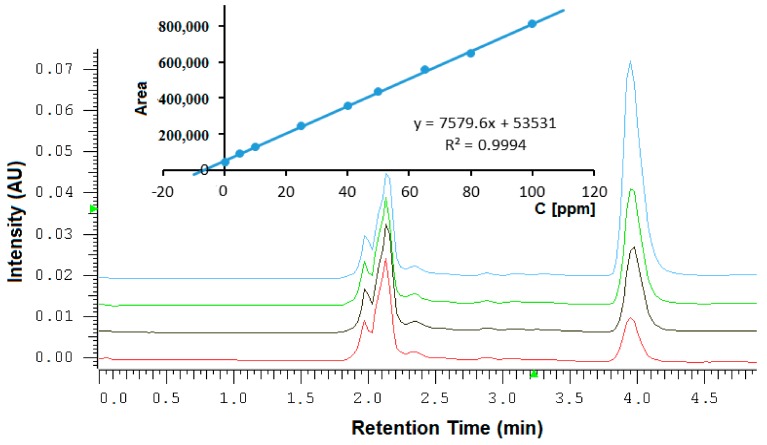
Chromatograms of saliva sample spiked in different standard concentrations (0 -red line, 5 mg L^−1^-black line, 10 mg L^−1^-green line, 25 mg L^−1^-blue line). Insert shows the regression line for this sample performing by the standard addition method. Measured the concentration of the unspiked sample is 7.06 mg L^−1^. Considering sample dilution, the final concentration of salivary thiocyanate is 56.50 mg L^−1^, respectively.

The method was applied to thiocyanate quantitation of saliva samples of 24 healthy volunteers. Salivary thiocyanate measurements were calibrated according to the standard addition method. Thus, thiocyanate concentrations were calculated from an eight-point calibration line based upon the addition of various concentrations of exogenous analyte (5–100 mg L^−1^). The peak area for each thiocyanate concentration was plotted against the nominal thiocyanate concentrations. Due to the presence of endogenous thiocyanate in saliva, such regression resulted in negative *x*-axis intercepts, which were used to calculate the concentration of endogenous thiocyanate taking into account sample dilution. The data were analyzed by least-squares linear regression. The obtained standard curves were linear in the tested calibration range resulted in regression coefficients of R^2^ = 0.99. A representative chromatogram of thiocyanate spiked in different standard concentration is shown in Figure 3. The thiocyanate peak in the chromatogram was eluted at retention time 3.95 min. Peak symmetry (As) was 1.18 and the theoretical plate number (*n*) was 4361. The peak was very well separated from other sample components eluted at the beginning of the chromatogram. The proposed system showed the ability to assess the analyte for the presence of various matrix components within shorter than 5 minutes. Thus, the method can be classified as specific. Calibration data for each sample are collected in Table 3.

**Figure 4:**
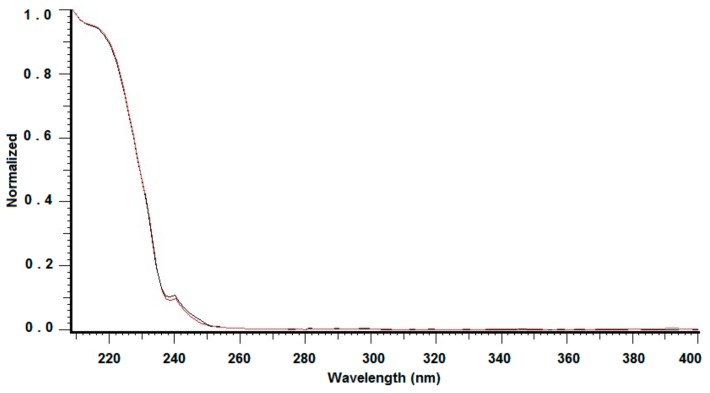
The SCN^−^ peak identification by spectral matching. The red line represents standard and the black line represents investigated analyte.

The identification of SCN^−^ peak performed by the UV/Vis photodiode array detector, combined with data acquisition software, is presented in Figure 4. The spectrum of the selected peak is compared against the reference spectrum of standard. The similarity index 0.9989 indicates the closeness of the match.

Table 4 contains the calculated data together with statistics. The thiocyanate content was calculated as for the standard addition method, i.e., sample level equals intercept/slope, with a calculated margin of error.

Table 5 shows the final thiocyanate concentration in human saliva of non-smokers, tobacco smokers, and e-cigarette smokers which were calculated multiplying the measured value by the sample dilution factor.

In order to compare the variance for the results obtained in the examined groups, the F-Snedecor test was used. Only the variance values calculated for the group of tobacco smokers and non-smokers differed statistically significantly. Using Student’s *t*-test and Cochran-Cox test, it was found that the average concentration of thiocyanates in the saliva of non-smokers and smokers (Student’s *t*-test t (8.83) > t_critical_ (2.14) and non-smokers and smokers of e-cigarettes (Cochran-Cox test) differ statistically significantly c (6.69) > c_critical_ (2.36). In contrast, differences in thiocyanate concentrations determined for both groups of smokers were not statistically significant (Student’s *t* test). For these comparing groups calculated the value of t (1.63) is smaller than the critical value (2.15) for seven degrees of freedom and for alpha = 0.05 (a 95% probability level).

## 3. Materials and Methods

### 3.1. Materials 

Deionized water to prepare all the aqueous solutions was purchased by ULTRAPURE Millipore Direct-Q 3UV-R (Merck, Darmstadt, Germany) of the resistivity 18.2 MΩ cm. Sodium chloride was from POCH (Gliwice, Poland). A stock standard solution of potassium thiocyanate (200 mg L^−1^) was prepared from potassium thiocyanate (Merck, Darmstadt, Germany). The working standard solutions were prepared immediately before use.

### 3.2. Demographic Characteristic

The study comprised 24 subjects, of which eight had a habit of tobacco smoking, eight had a habit of e-cigarette smoking, and eight smoked neither tobacco nor e-cigarette and comprised the healthy control group. The work has been carried out in accordance with The Code of Ethics of the World Medical Association (Declaration of Helsinki) for experiments involving humans. All the persons were in the age group of 40 to 45 years. Saliva was collected by the spitting method. Unstimulated whole saliva was analyzed immediately or after storage at room temperature or refrigerator at −18 °C and processed within 48 h.

### 3.3. HPLC Analysis and Characterization

The column used in the study was a IAM.PC.DD2 Regis HPLC (4.6 × 150 mm, 10 µm, pore size: 300 Å) from Agilent Technologies (Santa Clara, CA, USA). A mixture of inorganic salt (NaCl) in water was used as mobile phase and its flow rate was set at 1 mL min^−1^. The IAM.PC.DD2 packing is able to tolerate mobile phases between pH levels of 7.0 and 7.5. Therefore, the ionic strength of the mobile phase can be changed remaining pH at a neutral level resulting in longer column life. The oven temperature was set at 25 °C and the detection was performed at 210 nm. A HPLC (LaChrom HPLC Merck Hitachi, Germany) system equipped with a diode array detector (L-7455), pump (L-7100), interface (D-7000), and solvent degasser (L-7612), column oven (L-7350) was used. The mobile phases were filtered through a Nylon 66 membrane filter (0.45 µm) Whatman (Maidstone, England) using a filtration apparatus.

#### 3.3.1. Mobile Phase Composition

The influence of NaCl concentration in the mobile phase on the thiocyanate retention, the peak symmetry factor, and the system efficiency (N) was studied in the range of 1 to 20 mM (1, 5, 10, 15, 20 mM) at a flow rate of 0.5 mL/min to enhance retention, and injected volume 10 μL.

#### 3.3.2. The Sample Loading Capacity of Thiocyanate

The detrimental effect of sample overloading on peak width and its shape was studied by injection 10 µL of increasing sample concentration (10; 15; 25; 50; 80; 100; 150; 200 mg L^−1^). The volume overload was examined by injection an increasing volume from 3 to 20 µL, with the concentration at a constant level of 2 mg L^−1^. Analysis has been accomplished at optimized mobile phase composition (20 mM NaCl) and the flow rate of 1 mL min^−1^.

#### 3.3.3. Detection Wavelength and DAD Peak Identification

The maximum wavelength was established basing on absorption spectrum elaborated on for examined compound in the range from 200 nm to 400 nm. Further experiments with photodiode array detection were performed at analytical wavelength of 210 nm. The chromatographic peak identification was based on the comparison the retention times and UV spectra of isolated SCN^−^ and the standard. To identify the compounds, overlaid spectra of isolated peaks and appropriate standards were performed. The agreement of compared spectra was always higher than 0.995. Verification of the peak homogeneity was proved by the coincidence of UV spectra taken at upslope, apex, and downslope of the eluting peak.

#### 3.3.4. Data Analysis

All calculations were performed using the D-7000 HSM software. To calculate N-the number of theoretical plates according to USP standards, the following equation was used: N = 16(RT/W)2, where RT = the retention time and W = the peak width obtained by drawing tangents to each side of the peak and calculating the distance between the two points where the tangents meet the baseline. To calculate asymmetry, the following equation was used: As = 1/2(1 + B/A), where A and B are evaluated at a 5% peak height of an appropriate peak.

### 3.4. Thiocyanate Spiked Saliva Sample Preparation

Saliva from non-smokers (*n* = 8), tobacco smokers (*n* = 8), and e-cigarette smokers (*n* = 8) were collected directly from healthy volunteers, in sterile containers. For every donor, aliquots (6 mL) were immediately transferred into 15 mL vials. The samples were prepared as follows: 6 mL of the fresh saliva was mixed with 6 mL of water. After that, the mixtures were heated at 100 °C for 15 min, and then centrifuged at 4000 × *g* for 60 min. A volume of the clear supernatant (1 mL) of the supernatant was mixed with different volumes of a 200 mg L^−1^ aqueous solution of KSCN, and then made up to 4 mL with deionized water in order to have eight different thiocyanate concentrations (5; 10; 25; 40; 50; 65; 80 and 100 mg L^−1^) and construct the calibration curves. A volume of the calibrators (5 µL) was injected into the HPLC system.

### 3.5. Data Analysis

Linear regression analysis was performed by using Microsoft Excel 2010. Experimental values were expressed as the mean ± standard deviation (S.D.). The differences between groups were analyzed by the *t*-test, the Cochran-Cox, the F-Snedecor-test. Calculations were made in accordance with the literature [48]. The difference between two groups was judged to be statistically significant when *p* < 0.05.

## 4. Conclusions

The measurement of thiocyanate concentration in saliva is widely recognized as a non-invasive method for assessing exposure to nicotine smoke. Salivary thiocyanate is responsible for neurological and endocrine disorders; therefore, the development of a fast and reliable method for its determination is important from a clinical diagnosis point of view. The study presented is the first to assess and compare SCN^−^ levels in the saliva of e-cigarette smokers compared to non-smokers and smokers. This novel HPLC/IAM/DAD method represents a valuable tool for screening thiocyanate levels in routine diagnostics. The measured level of SCN^−^ was highly variable, with the highest levels observed in e-cigarette smokers. The level of SCN^−^ was even 121−244 mg L^−1^. This level was statistically significantly higher compared to the control group including non-smokers whose measured content ranged from 33−79 mg L^−1^. SCN^−^ levels in e-cigarette smokers turned out to be surprisingly high compared to the level measured for tobacco smokers which were in the range of 121−187 mg L^−1^. This is valuable data considering the fact that e-cigarette has gained recent widespread popularity. The proposed method utilizing HPLC on phosphatidylcholine column and UV detection can be suitable for further epidemiological studies. Where does SCN^−^ come from in e-cigarettes? This is difficult question to answer. This problem requires extensive study of the influence of components of e-cigarette liquids, temperature, and humidity to generate SCN^−^. Bourdoux [49] suggested previously that glucosinolates in the plants might contribute “per se” to an increased in levels of thiocyanates without the need to detoxify cyanide. Therefore, diet should be additionally controlled for to estimate the real toxicity of e-cigarettes in future studies.

## Figures and Tables

**Table 1:** Recovery of thiocyanate and its coefficient of variation (CV).

Concentration (mg L^−1^)	Recovery (%)	CV (%)
5	90.04	4.56
50	95.62	2.49
100	97.80	1.82

**Table 2:** Stability (%) of thiocyanate (SCN^−^) in saliva samples storage at room temperature and at refrigerator (6, 24, 48 h) (*n* = 5).

Sample (Room Temperature)	Contents Measured [mg L^−1^] Mean ± S.D.	Relative Bias * (%)	Sample (Refrigerator)	Contents Measured [mg L^−1^] Mean ± S.D.	Relative Bias * (%)
a	56.50 (±0.05)	-	a	61.48 (±0.04)	-
b	55.01 (±0.07)	−2.64	b	57.10 (±0.03)	−7.12
c	52.98 (±0.07)	−6.23	c	56.73 (±0.02)	−7.72
d	49.44 (±0.03)	−12.50	d	53.30 (±0.06)	−13.30

^a^ immediately, ^b^ after 6 h. ^c^ after 24 h ^d^ after 48 h of storage. * Relative bias (%) = (value measured after storage − value measured immediately after donation) × 100/value measured immediately after donation. Abbreviation S.D. indicates for the standard deviation value.

**Table 3:** Calibration data (*n* = 8).

Linear Regression Equation (y = ax + b)				
Slope (a ± s_a_)	Intercept (b ± s_b_)	Correlation Coefficient (R^2^)	the Standard Error of Estimate (s_e_)	Fisher F Statistic (F)
Nonsmokers				
7765.25 ± 114.21	32065.34 ± 6076.07	0.9984	11334.56	4623.15
7300.85 ± 92.94	72539.61 ± 4944.84	0.9988	9224.32	6170.42
7958.12 ± 128.84	42278.48 ± 6854.81	0.9981	12787.25	3815.06
7595.09 ± 135.17	41417.86 ± 7191.65	0.9978	13415.62	3157.04
7780.22 ± 65.14	69607.38 ± 3465.93	0.9995	6465.51	14263.12
8002.64 ± 70.26	60983.62 ± 3737.99	0.9995	6973.02	12973.60
7921.36 ± 104.08	73983.28 ± 5537.50	0.9988	10329.89	5792.21
8073.65 ± 33.28	67012.04 ± 1770.70	0.9998	3303.15	58846.43
Tobacco Smokers				
7809.52 ± 65.50	118360.34 ± 3484.88	0.9995	6500.86	14214.89
7775.82 ± 44.58	118561.55 ± 2372.04	0.9998	4424.92	30417.55
7971.41 ± 39.05	136039.10 ± 2077.91	0.9998	3876.22	41657.09
7803.99 ± 135.38	182913.64 ± 7202.95	0.9979	13436.71	3322.65
6874.31 ± 111.77	137752.34 ± 5946.72	0.9981	11093.28	3782.46
6958.16 ± 73.98	142008.39 ± 3935.97	0.9992	7342.35	8846.17
7983.35 ± 56.65	137911.51 ± 3014.14	0.9996	5622.72	19856.98
7930.19 ± 63.41	156159.97 ± 3373.81	0.9995	6293.65	15638.61
e-Cigarette Smokers				
7778.52 ± 24.39	183659.60 ± 1297.89	0.9999	2421.14	101668.97
7890.01 ± 130.91	200175.71 ± 6965.19	0.9981	12993.17	3632.13
7539.40 ± 83.05	121734.70 ± 4418.33	0.9992	8242.15	8241.93
7727.04 ± 130.85	117098.02 ± 6962.03	0.9980	12987.27	3486.79
7857.74 ± 93.31	206405.09 ± 4964.52	0.9990	9261.04	7091.07
7677.98 ± 86.58	165317.86 ± 4606.57	0.9991	8593.30	7863.41
7868.81 ± 34.81	140711.23 ± 1851.83	0.9999	3454.50	51107.37
7846.51 ± 45.22	239426.03 ± 2405.84	0.9998	4487.95	30108.72

**Table 4:** A point prediction and prediction interval for the thiocyanate concentration in saliva samples and given Y (0) value in a simple regression.

Std. Error of Regression	Point Prediction	t (alpha10%)	St. Error of Prediction	Margin of Error	Lower Bound	Upper Bound
**nonsmokers**						
1.5738	4.1293	0.9236	1.8407	1.7	2.4293	5.8293
1.2903	9.9358	0.9236	1.545	1.4269	8.5089	11.3627
1.7287	5.3126	0.9236	2.0312	1.876	3.4366	7.1886
1.8347	5.4532	0.9236	2.1569	1.9921	3.4611	7.4453
0.849	8.9467	0.9236	1.0124	0.9351	8.0116	9.8818
0.9383	7.6204	0.9236	1.1129	1.0279	6.5925	8.6483
1.3886	9.3397	0.9236	1.6585	1.5318	7.8079	10.8715
0.4412	8.3001	0.9236	0.5247	0.4846	7.8155	8.7847
**tobacco smokers**						
0.7833	15.1559	0.9236	0.9591	0.8859	14.27	16.0418
0.517	15.2475	0.9236	0.6334	0.585	14.66247	15.83247
0.5233	17.0659	0.9236	0.6462	0.5969	16.46898	17.66278
1.8086	23.4385	0.9236	2.299	2.1233	21.31518	25.56178
1.741	20.0387	0.9236	2.1787	2.0122	18.02652	22.05092
1.057	20.4089	0.9236	1.3252	1.224	19.1849	21.6329
0.7528	17.2749	0.9236	0.9306	0.8595	16.41539	18.13439
0.7707	19.6918	0.9236	0.9632	0.8896	18.80223	20.58143
**e-cigarette smokers**						
0.3359	23.6111	0.9236	0.4275	0.3948	23.2163	24.0059
1.5938	25.3708	0.9236	2.0445	1.8883	23.4825	27.2591
1.1385	16.1465	0.9236	1.4002	1.2932	14.8533	17.4397
1.7981	15.1543	0.9236	2.2014	2.0332	13.1211	17.1875
1.2701	26.2677	0.9236	1.6363	1.5113	24.7564	27.7790
1.11	21.5314	0.9236	1.3988	1.2919	20.2395	22.8233
0.4709	17.8821	0.9236	0.5837	0.5391	17.3430	18.4212
0.6177	30.5137	0.9236	0.8123	0.7502	29.7635	31.2639

Std. Error—the standard error. Calculations have been made assuming the desired prediction level: 90%, alpha 10%. The standard error for prediction value was calculated using the following equation: $SE_{pred.}=s\sqrt{(1+\frac{1}{n}}+\frac{{\left(x-\bar{x}\right)}^{2}}{\left(n-1\right)s_{x}^{2}})$, where *n* stands for the number of total observations, *s* is for the standard error of regression, *s_x_* is the standard deviation of the measured values, $\bar{x}$ is average value of measured observations, margin of error was calculated by multiplying *t* and *SE_pred._*, and $t_{\left(1-\frac{\alpha }{2},n-2\right)}$ was estimated for 10% of alpha and *n*-2 of the degree of freedom.

**Table 5:** Thiocyanate levels in human saliva of non-smokers, tabacco smokers, and e-cigarette smokers. Mean values of healthy donors are expressed in mg L^−1^, S.D. is the standard deviation.

Nonsmokers		Tobacco Smokers		e-Cigarette Smokers	
Mean ± S.D. [mg L^−1^]	Concentration Range [mg L^−1^]	Mean ± S.D. [mg L^−1^]	Concentration Range [mg L^−1^]	Mean ± S.D. [mg L^−1^]	Concentration Range [mg L^−1^]
59.04 ± 17.18	33.03−79.49	148.32 ± 22.82	121.25−187.54	176.46 ± 43.09	121.24−244.11
